# Uncovering the mechanism of Radix Paeoniae Alba in the treatment of restless legs syndrome based on network pharmacology and molecular docking

**DOI:** 10.1097/MD.0000000000031791

**Published:** 2022-11-18

**Authors:** Jun Liu, Suxian Liu, Liansheng Hao, Fangfang Liu, Shengkai Mu, Tengteng Wang

**Affiliations:** a Department of Gastroenterology, Longhua Hospital, Shanghai University of Traditional Chinese Medicine, Shanghai, China; b Institute of Digestive Diseases, Longhua Hospital, Shanghai University of Traditional Chinese Medicine, Shanghai, China; c Department 2 of Bone Trauma, Liaocheng Hospital of Traditional Chinese Medicine, Liaocheng, China; d Longhua Hospital, Shanghai University of Traditional Chinese Medicine, Shanghai, China; e Department of Acupuncture, Longhua Hospital, Shanghai University of Traditional Chinese Medicine, Shanghai, China.

**Keywords:** mechanism, molecular docking, network pharmacology, Radix Paeoniae Alba, restless legs syndrome

## Abstract

Restless legs syndrome (RLS) is a neurological motor disorder with a high prevalence. The treatment efficacy of RLS is unsatisfactory. Radix Paeoniae Alba (RPA) can effectively treat RLS symptoms such as the discomfort of the legs. RPA has great potential for the development of new medications for RLS. Hence, we explored the mechanism of RPA in the treatment of RLS using network pharmacology and molecular docking. The active components and targets of RPA were obtained from the Traditional Chinese Medicine System Pharmacology database and analysis platform and PharmMapper platform. The RLS-related targets were found in GeneCards, OMIM, DrugBank, and DisGeNET databases. The overlapping targets of RPA and RLS were then collected. The “active components-overlapping targets” network was built, and network topology analysis was performed. Furthermore, Cytoscape 3.9.1 software was used to screen the key components of RPA in the treatment of RLS. Protein-protein interaction was performed using the Search Tool for the Retrieval of Interacting Genes. The gene ontology functions and Kyoto Encyclopedia of Genes and Genomes signaling pathways were analyzed using ClusterProfiler, PathView, and other R packages to reveal the main mechanism of RPA in treating RLS. Component and protein structures were downloaded from the Traditional Chinese Medicine System Pharmacology and Protein Data Bank databases, respectively. The AutoDock 4.2.6 software was used for molecular docking. A total of 12 active components and 109 targets of RPA, as well as 2387 RLS-related targets, were collected. Following that, 47 overlapping targets were obtained. Furthermore, 5 key components and 12 core targets were screened. The results of gene ontology functions were as follows: 2368 biological processes, 264 molecular functions, and 164 cellular components. A total of 207 Kyoto Encyclopedia of Genes and Genomes signaling pathways were obtained, including the lipid and atherosclerosis pathway, the endocrine resistance pathway, the prolactin signaling pathway, and the IL-17 signaling pathway. The components and the core targets completed molecular docking stably. RPA has multi-component, multi-target, and multi-pathway characteristics in treating RLS, which could provide a basis for future research and improve clinical efficacy.

## 1. Introduction

Restless legs syndrome (RLS) is a common neurological motor disorder.^[1,2]^ The main symptoms include sensory abnormalities of both calves, thighs, trunks, and arms (calves are the most affected body parts), which frequently occur at rest, especially at night.^[1,3–8]^ Patients are usually forced to move the affected parts to alleviate their discomfort.^[2,9]^ RLS can cause or coexist with cardiovascular, metabolic, sleep, and mental disorders (such as anxiety and depression)^[1,2,10–12]^ and reduce quality of life.^[5,11,13–16]^ The prevalence of RLS has been estimated to range from 4% to 29%,^[11]^ and it has attracted wide attention in recent years. The etiology and pathogenesis of RLS still remain unclear. Dopamine agonists and gabapentinoids are currently used as first-line medications. However, their therapeutic effects are unsatisfactory.^[5,12]^

In Traditional Chinese Medicine (TCM), RLS is classified as “arthralgia” and “tibial acid.” In clinics, Radix Paeoniae Alba (RPA) can effectively relieve the discomfort of the legs and treat RLS. However, there are few studies on the efficacy and mechanism of RPA in treating RLS. RPA can effectively treat related brain disorder-related diseases, such as Parkinson’s disease (PD). PD is a neurological disorder in which there are disturbances in the movement including resting tremors, rigidity, bradykinesia or akinesia.^[17]^ The pathogenesis of PD involves dysfunction of dopaminergic neurons, iron metabolism disorders, oxidative stress, and abnormal immune reactions.^[17]^ Total glucosides of paeony (TGP) of RPA were extracted from dried roots of RPA, and paeoniflorin was the main active compound of TGP.^[18]^ TGP could significantly increase the level of dopamine and its metabolites in striatum of PD mice and improve the motor coordination.^[19]^ Paeoniflorin had neuroprotective effects and ameliorated motor dysfunction in both PD rats^[20]^ and mouse models.^[21–23]^ Dopaminergic dysfunction,^[24,25]^ iron deficiency,^[24–26]^ oxidative stress,^[27]^ and immunological alterations^[28]^ are also associated with the pathogenesis of RLS. Therefore, RPA should be effective in the treatment of RLS and the mechanism is worth to be elucidated.

**Figure 1.**
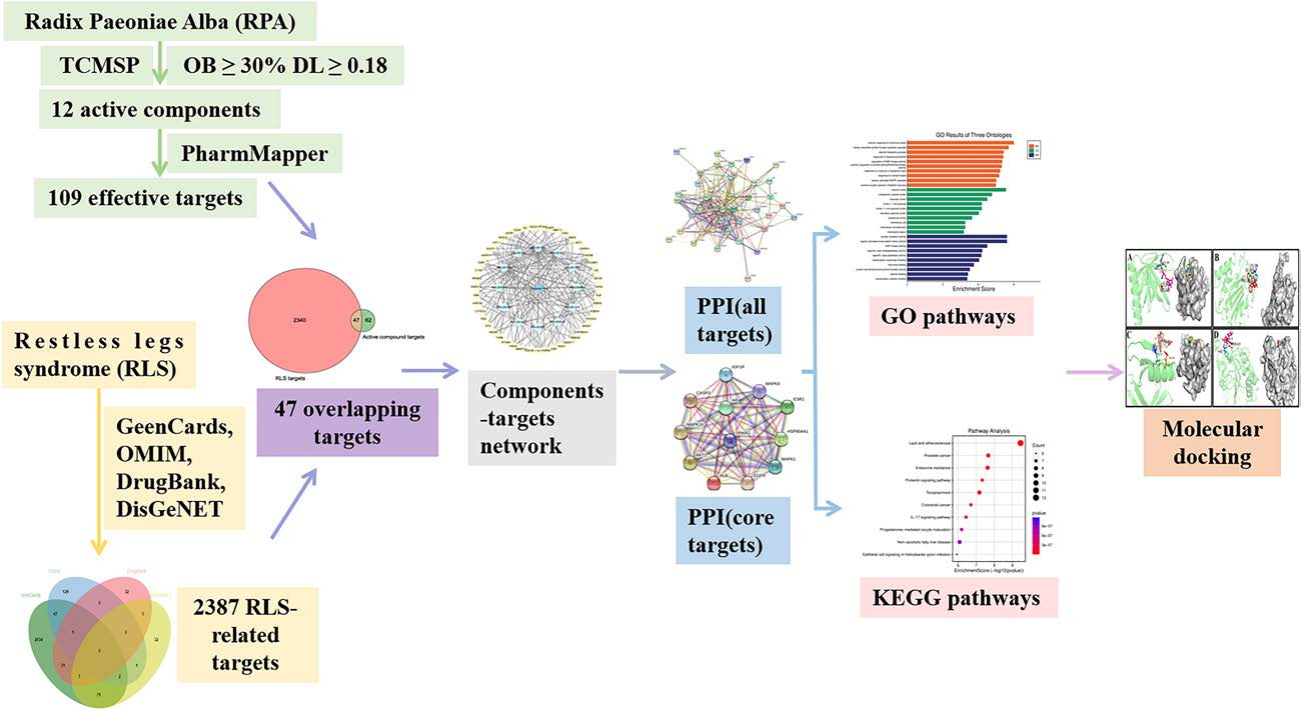
The workflow of this study. The active components and targets of RPA, as well as RLS-related targets were obtained. The overlapping targets of RPA and RLS were collected. The “components-targets” network was then built. Furthermore, the PPI network was constructed. The GO functions and KEGG signaling pathways were analyzed. Finally, the molecular docking was performed. GO = gene ontology, KEGG = Kyoto Encyclopedia of Genes and Genomes, PPI = protein-protein interaction, RLS = restless legs syndrome, RPA = Radix Paeoniae Alba.

Network pharmacology can reveal the synergistic effect of multi-molecule medications through big data analysis, providing a practical basis and effective way for the research and innovation of TCM. In this study, the active components, core targets, and main signaling pathways of RPA in the treatment of RLS were screened using network pharmacology. The binding of components and targets was performed through molecular docking. The mechanism of RPA in treating RLS was revealed, providing the basis for future research and clinical applications. The workflow of this study is depicted in Figure 1.

## 2. Methods

### 2.1. Screening active components of RPA

The components of RPA were collected in the Traditional Chinese Medicine System Pharmacology Database (TCMSP, https://old.tcmsp-e.com/tcmsp.php).^[29]^ Based on the absorption, distribution, metabolism, and excretion (ADME) parameters provided by TCMSP, oral bioavailability ≥ 30% and drug likeness ≥ 0.18 were set as the limiting conditions for screening the active components. Furthermore, the components of RPA were supplemented by retrieving the published papers in CNKI (https://www.cnki.net/) and PubMed. The chemical structures of these components were obtained from PubChem (https://pubchem.ncbi.nlm.nih.gov/)^[30]^ for Swiss ADME prediction,^[31]^ which was required to be equal to HIGH and at least 2 terms of drug likeness were YES.^[32]^

### 2.2. Gathering potential targets of active components of RPA

The mol2 files of active components of RPA were downloaded from the TCMSP database and uploaded to the PharmMapper server to search for potential targets.^[33]^ The species were set as human protein, and the parameter value was set as default. Excel and UniProt database (https://www.uniprot.org/)^[34]^ were used to merge, deduplicate, and standardize the data obtained from the targets. The targets that were not “reviewed” were deleted.

### 2.3. Collecting RLS-related targets

The search term “restless legs syndrome” was used to obtain the targets of RLS in GeneCards (https://www.genecards.org/),^[35]^ OMIM (https://www.omim.org/),^[36]^ DrugBank (https://go.drugbank.com/),^[37]^ and DisGeNET databases (https://www.disgenet.org/).^[38]^ The results were merged, deduplicated, and standardized.

### 2.4. Obtaining the overlapping targets of RPA and RLS

The overlapping targets of RPA and RLS were obtained after being processed by Excel and the Bioinformatics website (http://www.bioinformatics.com.cn/), which were the potential targets of RPA in treating RLS.

### 2.5. Construction of “active components-overlapping targets” network and screening key active components

The “active components-overlapping targets” network was built using Cytoscape 3.9.1.^[38]^ The network topology was examined using its own Network Analyzer analysis tool, and the key components of RPA involved in treating RLS were screened based on the topological parameter degree.

### 2.6. Construction of the protein-protein interaction (PPI) network

To systematically explore the complicated network synergy between the potential targets of RPA and RLS, the overlapping targets were imported into the Search Tool for the Retrieval of Interacting Genes (https://string-db.org/).^[39]^ Moreover, the PPI network with the species limited to “Homo sapiens” was built. Furthermore, the minimum interaction threshold was set to medium confidence (0.4) for analysis. The PPI network was then built, and the network topology was analyzed. The core targets with close interaction were screened based on the degree of the topological parameter.

### 2.7. Gene ontology (GO) and Kyoto Encyclopedia of Genes and Genomes (KEGG) enrichment analysis

The overlapping targets were imported into R packages such as clusterProfiler^[39]^ and pathview^[40]^ to perform the GO and KEGG enrichment analysis. The GO function included biological process (BP), molecular function (MF), and cellular component (CC). The “gene-pathway” network was built to visualize the primary mechanism of RPA in the treatment of RLS.

### 2.8. Molecular docking

The structure of components and proteins were obtained from the TCMSP and Protein Data Bank databases, respectively (https://www.rcsb.org/).^[41]^ The molecular docking of the components and targets was then performed using AutoDock4.2.6 software. Finally, the results were visualized using PyMoL 2.5.0 software.

### 2.9. Ethical review

This article didn’t contain any studies with human participants or animals performed by any of the authors. Hence, ethical review was not necessary.

## 3. Results

### 3.1. Active components and targets of RPA

**Figure 2.**
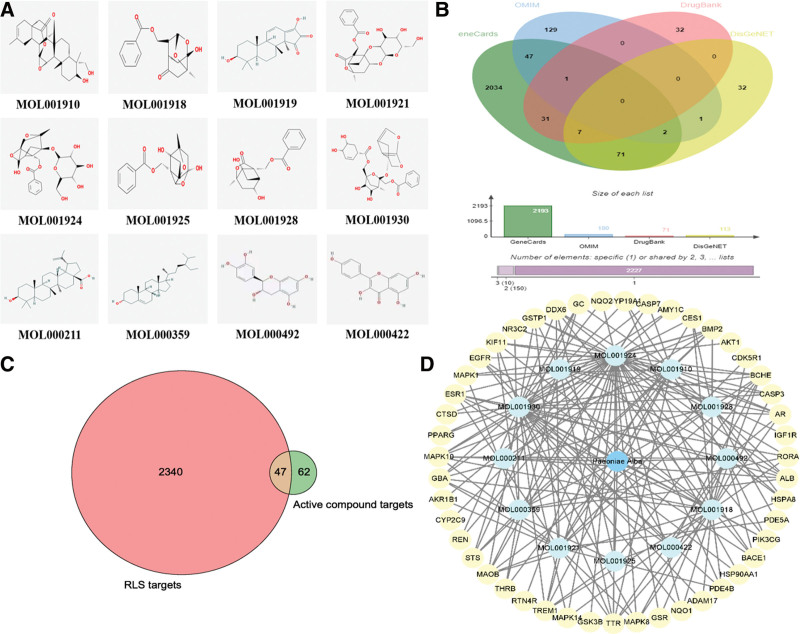
The active components and targets. (A) The chemical structures of 12 active components of RPA. (B) The RLS-related targets. The green, blue, pink, and yellow ellipse represented the GeneCards, OMIM, DrugBank and DisGeNET databases, respectively. (C) The RPA-RLS overlapping targets. The pink, green, and brown circle represented the targets of RPA, the RLS-related targets and the overlapping targets, respectively. (D) The “active components-overlapping targets” network had 60 nodes and 174 edges. The blue, light blue, and yellow circular nodes represented RPA, the active components, and the overlapping targets, respectively. The target relationships were represented by edges between nodes. RLS = restless legs syndrome, RPA = Radix Paeoniae Alba.

In total, 85 components of RPA were collected from the TCMSP database and published articles. Following ADME parameter screening and Swiss ADME prediction,^[31]^ 12 active components of RPA were obtained (Table 1). Figure 2A depicts the chemical structures of these components. Furthermore, 109 potential targets of 12 active components were obtained from the PharmMapper database.

**Table 1:** The active components of Radix Paeoniae Alba.

Mol ID	Molecule name	Oral bioavailability (%)	Drug likeness
MOL001910	11alpha,12alpha-epoxy-3beta-23-dihydroxy-30-norolean-20-en-28,12beta-olide	64.77	0.38
MOL001918	paeoniflorgenone	87.59	0.37
MOL001919	(3S,5R,8R,9R,10S,14S)-3,17-dihydroxy-4,4,8,10,14-pentamethyl-2,3,5,6,7,9-hexahydro-1H-cyclopenta^9^phenanthrene-15,16-dione	43.56	0.53
MOL001921	Lactiflorin	49.12	0.8
MOL001924	Paeoniflorin	53.87	0.79
MOL001925	paeoniflorin_qt	68.18	0.4
MOL001928	albiflorin_qt	66.64	0.33
MOL001930	benzoyl paeoniflorin	31.27	0.75
MOL000211	Mairin	55.38	0.78
MOL000359	Sitosterol	36.91	0.75
MOL000492	(+)-catechin	54.83	0.24
MOL000422	Kaempferol	41.88	0.24

### 3.2. The RLS-related targets

Using the keyword “restless legs syndrome,” 2193, 180, 71, and 113 targets associated with RLS were collected from the GeneCards, OMIM, DrugBank, and DisGeNET databases, respectively (Fig. 2B). Finally, 2387 RLS-related targets were obtained after removing the duplicates.

### 3.3. The overlapping targets of RPA and RLS

A total of 47 overlapping targets of the components and the disease were identified as potential targets of RPA in the treatment of RLS (Fig. 2C; Table 2).

**Table 2:** The overlapping targets of Radix Paeoniae Alba and Restless legs syndrome.

Gene name	Protein name	Uniprot ID	Degree
ALB	Albumin	P02768	32
AR	Androgen receptor	P10275	17
BCHE	Butyrylcholinesterase	P06276	4
BMP2	Bone morphogenetic protein 2	P12643	8
CASP7	Caspase 7	P55210	7
GC	Vitamin D-binding protein	P02774	4
KIF11	Kinesin-like protein KIF11	P52732	0
MAPK1	Mitogen-activated protein kinase 1	P28482	15
MAPK10	Mitogen-activated protein kinase 10	D6RBH2	7
STS	Steryl-sulfatase	P08842	3
THRB	Thyroid hormone receptor *β*	P10828	3
TREM1	Triggering receptor expressed on myeloid cells 1	Q38L15	0
TTR	Transthyretin	P02766	6
ADAM17	Disintegrin and metalloproteinase domain-containing protein 17	P78536	7
BACE1	Beta-secretase 1	P56817	10
CASP3	Caspase 3	P42574	24
CDK5R1	Cyclin-dependent kinase 5 activator 1	Q15078	5
CES1	Liver carboxylesterase 1	H3BSU0	3
CYP19A1	CYP19A1 protein	Q7Z471	9
DDX6	Probable ATP-dependent RNA helicase DDX 6	P26196	1
EGFR	Epidermal growth factor receptor	P00533	21
ESR1	Estrogen Receptor 1	P03372	21
GBA	Lysosomal acid glucosylceramidase	P04062	3
MAOB	Amine oxidase B	P27338	3
MAPK14	Mitogen-activated protein kinase 14	Q16539	16
MAPK8	Mitogen-activated protein kinase 8	C9JWQ4	16
NQO1	NAD(P)H dehydrogenase 1	P15559	8
PDE4B	cAMP-specific 3’,5’-cyclic phosphodiesterase 4B	Q07343	0
PIK3CG	Phosphatidylinositol 4,5-bisphosphate 3-kinase catalytic subunit gamma isoform	P48736	5
RORA	Nuclear receptor ROR-alpha	P35398	0
GSTP1	Glutathione S-transferase P1	P09211	10
AKR1B1	Aldo-keto reductase family 1 member B1	P15121	8
PDE5A	cGMP-specific 3’,5’-cyclic phosphodiesterase	O76074	3
AKT1	RAC-alpha serine/threonine-protein kinase	P31749	28
AMY1C	Alpha-amylase 1C	P0DTE8	1
CTSD	Cathepsin D	P07339	7
CYP2C9	Cytochrome P450 2C9	P11712	5
GSK3B	Glycogen synthase kinase-3 beta	P49841	11
GSR	Glutathione reductase, mitochondrial	P00390	9
HSP90AA1	Heat shock protein HSP 90-alpha	P07900	21
HSPA8	Heat shock cognate 71 kDa protein	P11142	11
IGF1R	Insulin-like growth factor 1 recepto	P08069	15
NQO2	Ribosyldihydronicotinamide dehydrogenase	P16083	0
NR3C2	Mineralocorticoid receptor	P08235	5
PPARG	Peroxisome proliferator-activated receptor gamma	P37231	17
REN	Renin	P00797	9
RTN4R	Reticulon-4 receptor	Q9BZR6	0

### 3.4. “Active components-overlapping targets” network and key active components

The data from 12 active components and 47 overlapping targets were imported into Cytoscape 3.9.1 software to build the “active components-overlapping targets” network (Fig. 2D). A total of 12 components were provided with a degree. The degree of 5 components was greater than ten, and these components were closely associated with RLS-related targets and might play a crucial role in the treatment of RLS (Table 3).

**Table 3:** The key active components of Radix Paeoniae Alba in the treatment of Restless legs syndrome.

Mol ID	MOL001924	MOL001930	MOL001918	MOL001921	MOL001910
Degree	39	32	24	15	13

### 3.5. The PPI network

**Figure 3.**
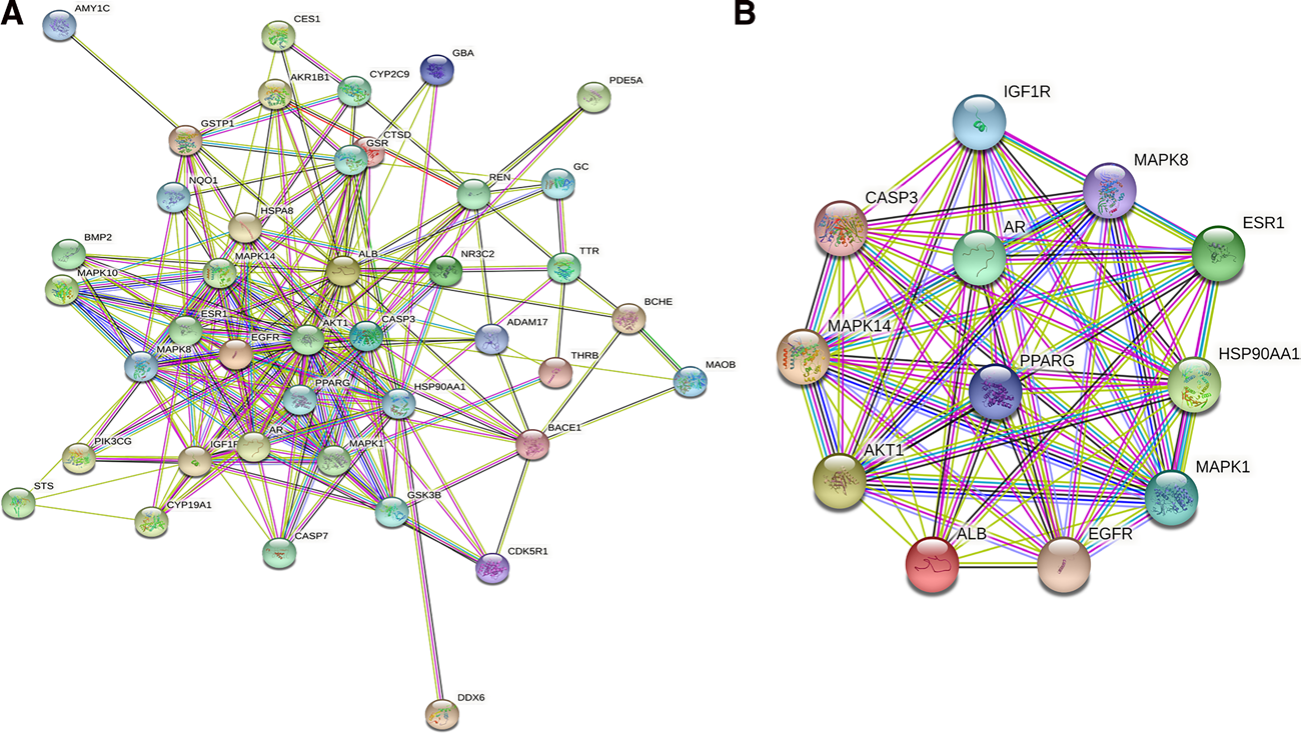
The PPI network. The nodes and edges represented the targets and their connection, respectively. (A) The PPI network of the overlapping targets with 47 nodes and 209 edges. (B) The PPI network of the core targets with 12 nodes and 64 edges. PPI = protein-protein interaction.

The PPI network of the overlapping targets contained 47 nodes and 209 edges (Fig. 3A). The nodes represented the targets, while the edges represented their connection. The median degree was 7, and the targets with a degree ≥ 2 times the median were the core targets. There were 12 nodes and 64 edges in the PPI network of the core targets (Fig. 3B; Table 4). These targets might be crucial in the mechanism of RPA in treating RLS.

**Table 4:** The core targets of Radix Paeoniae Alba in the treatment of Restless legs syndrome.

**Gene name**	ALB	AKT1	CASP3	EGFR	ESR1	HSP90AA1	AR	PPARG	MAPK14	MAPK8	IGF1R	MAPK1
**Degree**	32	28	24	21	21	21	17	17	16	16	15	15

### 3.6. GO enrichment analysis

#### 3.6.1. BP analysis.

**Figure 4.**
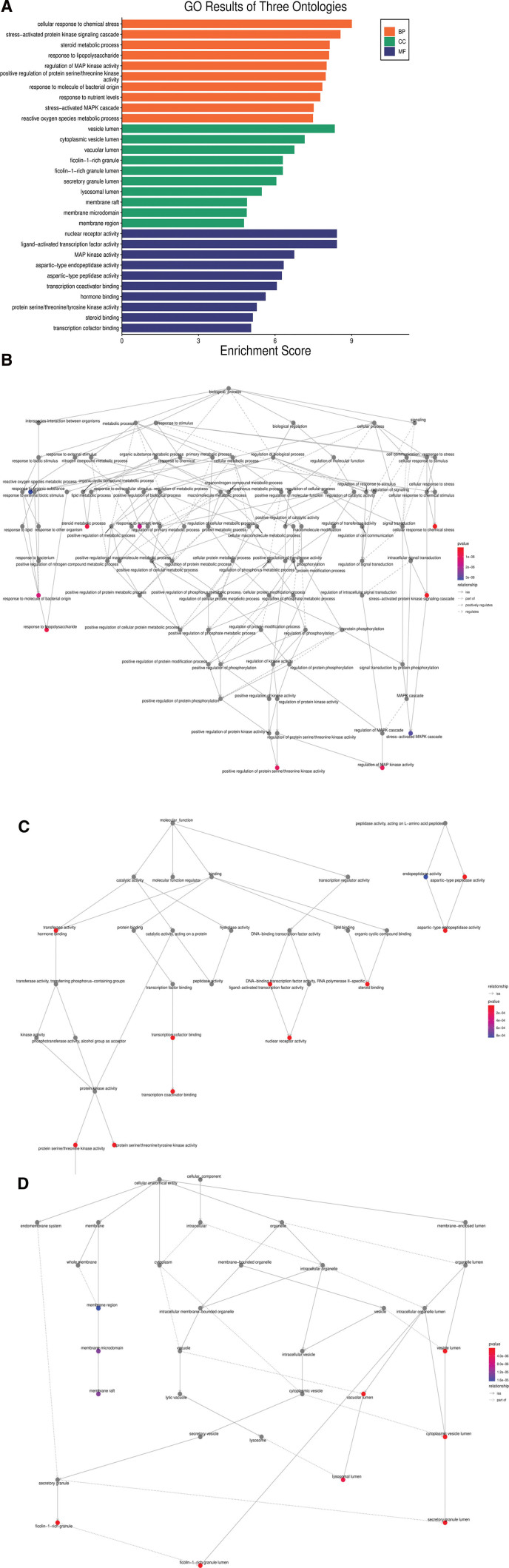
The results of GO analysis. (A) The top 10 enrichment results of BP, MF and CC. (B) The significance of BP functions and their connection. The nodes represented GO functions while the edges represented their connection. The color of nodes represented the *P* value. The redder the color, the lower the *P* value was. The red nodes represented the following functions: steroid metabolic process, regulation of MAP kinase activity, positive regulation of protein serine/threonine kinase activity, kinase signaling cascade, response to lipopolysaccharide, chemical stress, nutrient levels, and molecule of bacterial origin. (C) The significance of MF analysis and their connection. The red nodes represented the following functions: steroid binding, nuclear receptor activity, MAP kinase activity, ligand-activated transcription factor activity, aspartic-type endopeptidase activity, aspartic-type peptidase activity, transcription coactivator binding, hormone binding and protein serine/threonine/tyrosine kinase activity. (D) The significance of CC functions and their connection. The red nodes represented the following functions: vesicle lumen, vacuolar lumen, lysosomal lumen, ficolin-1-rich granule, ficolin-1-rich granule lumen, cytoplasmic vesicle lumen and secretory granule lumen. BP = biological process, CC = cellular component, GO = gene ontology, MF = molecular function.

There were 2368 BP enrichment results which primarily involved response to lipopolysaccharide, steroid metabolic process, chemical stress, and regulation of MAP kinase activity. The significance of these functions and the connection between them are depicted in Figure 4A and B. The higher the enrichment score and the smaller the *P* value, the more significant the function was. According to the enrichment score and *P* value, the top 10 enrichment results of BP were selected (Fig. 4A; Table 5).

**Table 5:** The top 10 biological process analysis.

ID	Biological process analysis	Gene names
GO:0062197	Cellular response to chemical stress	AKT1 AKR1B1 CASP3 NQO1 EGFR GSR MAPK1 MAPK8
GO:0070302	Stress-activated protein kinase signaling cascade	BMP2 EGFR GSTP1 IGF1R MAPK1
GO:0008202	Steroid metabolic process	AKR1B1 STS CES1 CYP2C9 CYP19A1 ESR1 GBA GC
GO:0031663	Response to lipopolysaccharide	AKT1 MAPK14 MAPK1
GO:0043405	Regulation of MAP kinase activity	BMP2 EGFR GBA GSTP1 PIK3CG PPARG PDE5A
GO:0071902	Positive regulation of protein serine/threonine kinase activity	AKT1 BMP2 EGFR PIK3CG ADAM17 PDE5A CDK5R1
GO:0002237	Response to molecule of bacterial origin	AKT1 CASP3 MAPK14 GSTP1 MAOB PDE4B MAPK1 MAPK8 REN ADAM17
GO:0031667	Response to nutrient levels	AKT1 ALB BCHE MAPK14 NQO1 GBA HSPA8 IGF1R PPARG MAPK1 MAPK8
GO:0051403	Stress-activated MAPK cascade	MAPK14 MAPK1 MAPK8 MAPK10
GO:0034614	Reactive oxygen species metabolic process	AKT1 NQO1 EGFR MAPK1 MAPK8

#### 3.6.2. MF analysis.

There were 264 MF enrichment results which primarily included hormone binding, MAP kinase activity, and steroid binding (Fig. 4A and C). The top 10 enrichment results of MF are shown in Figure 4 and Table 6.

**Table 6:** The top 10 molecular function analysis.

ID	Molecular function analysis	Gene names
GO:0004879	Nuclear receptor activity	AR ESR1 NR3C2 PPARG RORA THRB
GO:0098531	Ligand-activated transcription factor activity	AR ESR1 NR3C2 PPARG RORA THRB
GO:0004707	MAP kinase activity	MAPK14 MAPK1 MAPK8 MAPK10
GO:0004190	Aspartic-type endopeptidase activity	CASP3 CASP7 CTSD REN BACE1
GO:0070001	Aspartic-type peptidase activity	CASP3 CASP7 CTSD REN BACE1
GO:0001223	Transcription coactivator binding	AR ESR1 RORA THRB
GO:0042562	Hormone binding	AR EGFR IGF1R THRB TTR
GO:0004712	Protein serine/threonine/tyrosine kinase activity	AKT1 MAPK14 EGFR GSK3B IGF1R PIK3CG MAPK1 MAPK8 MAPK10
GO:0005496	Steroid binding	AR ESR1 GC NR3C2 RORA
GO:0008134	Transcription region	AR MAPK14 ESR1 GSK3B PPARG RORA THRB

#### 3.6.3. CC analysis.

There were 164 CC enrichment results which consisted of vesicle lumen, cytoplasmic vesicle lumen, vacuolar lumen, and others (Fig. 4A and D). Figure 4A and Table 6 represent the top 10 enrichment results of CC.

All the results of GO enrichment analysis suggested that a variety of biological processes were involved in the mechanism of RPA in treating RLS.

### 3.7. KEGG pathway enrichment analysis

**Figure 5.**
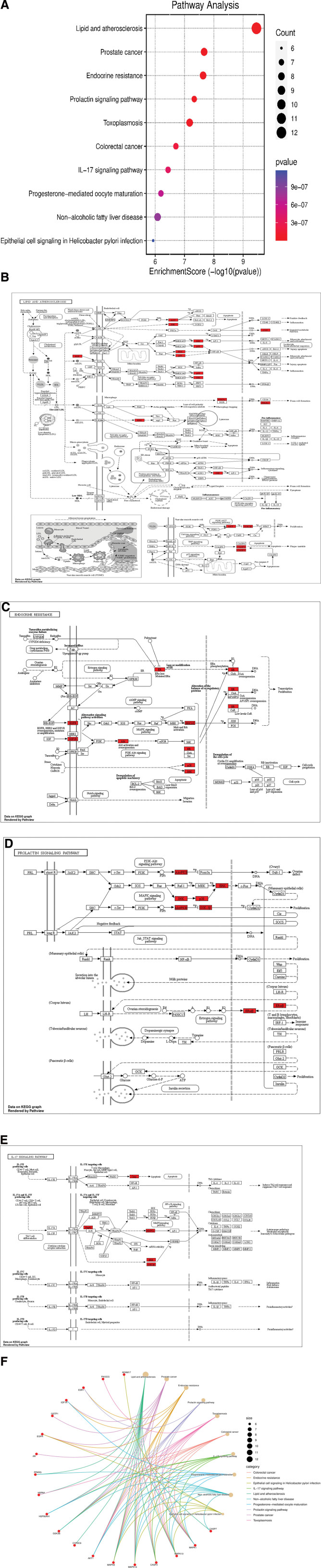
The pathway analysis. (A) The top 10 enrichment results of KEGG. The color of nodes represented the *P* value. The redder the color, the lower the *P* value was. The higher the enrichment score and the smaller the *P* value, the more significant the function was. (B) The lipid and atherosclerosis pathway. The proteins marked red in this pathway were closely associated with the overlapping targets of RPA and RLS (AKT, JNK, p38, ERK, CYP, HSP, PPAR*γ*, GSK3B, CASP3, and CASP7). (C) The endocrine resistance pathway. The proteins marked red were closely related to the overlapping targets (ER, JNK, p38, ERK1/2, AKT, EGFR, and IGF1R). (D) The prolactin signaling pathway. The proteins marked red were closely associated with the overlapping targets (AKT, JNK, p38, ERK, GSK3B, and ERA/B). (E) The IL-17 signaling pathway. The proteins marked red were closely associated with the overlapping targets (CASP, HSP90, MAPKS, ERK, and GSK3B). (F) The gene-pathway network. The red and brown nodes represented genes and pathways, respectively. The edges represented the connection between them. KEGG = Kyoto Encyclopedia of Genes and Genomes, RLS = restless legs syndrome, RPA = Radix Paeoniae Alba.

A total of 207 KEGG enrichment results were obtained, and the top 10 results were selected using enrichment score and *P* value (Fig. 5A). Many pathways, including lipid and atherosclerosis (Fig. 5B), endocrine resistance pathway (Fig. 5C), prolactin signaling pathway (Fig. 5D), IL-17 signaling pathway (Fig. 5E), and others were involved in the mechanism of RPA in treating RLS. The genes involved in the top 10 KEGG signaling pathways are listed in Table 7. The gene-pathway network (Fig. 5F) was built through the microbiot platform to visualize the relationship between the main target genes and the pathways of RPA in treating RLS.

**Table 7:** The top 10 cellular component analysis.

ID	Cellular component analysis	Gene names
GO:0031983	Vesicle lumen	ALB MAPK14 CTSD EGFR GSTP1 HSPA8 HSP90AA1 MAPK1 TTR BACE1
GO:0060205	Cytoplasmic vesicle lumen	ALB MAPK14 CTSD GSTP1 HSPA8 HSP90AA1 MAPK1 TTR BACE1
GO:0005775	Vacuolar lumen	CTSD GBA GC HSPA8 HSP90AA1 MAPK1 TTR
GO:0101002	Ficolin-1-rich granule	MAPK14 CTSD GSTP1 HSPA8 HSP90AA1 MAPK1
GO:1904813	Ficolin-1-rich granule lumen	MAPK14 CTSD GSTP1 HSPA8 HSP90AA1 MAPK1
GO:0034774	Secretory granule lumen	ALB MAPK14 CTSD GSTP1 HSPA8 HSP90AA1 MAPK1 TTR
GO:0043202	Lysosomal lumen	CTSD GBA GC HSPA8 HSP90AA1
GO:0045121	Membrane raft	CASP3 CTSD EGFR IGF1R MAPK1 ADAM17 BACE1 RTN4R
GO:0098857	Membrane microdomain	CASP3 CTSD EGFR IGF1R MAPK1 ADAM17 BACE1 RTN4R
GO:0098797	Membrane protein complex	BMP2 CASP3 EGFR IGF1R PDE4B

### 3.8. Molecular docking

**Figure 6.**
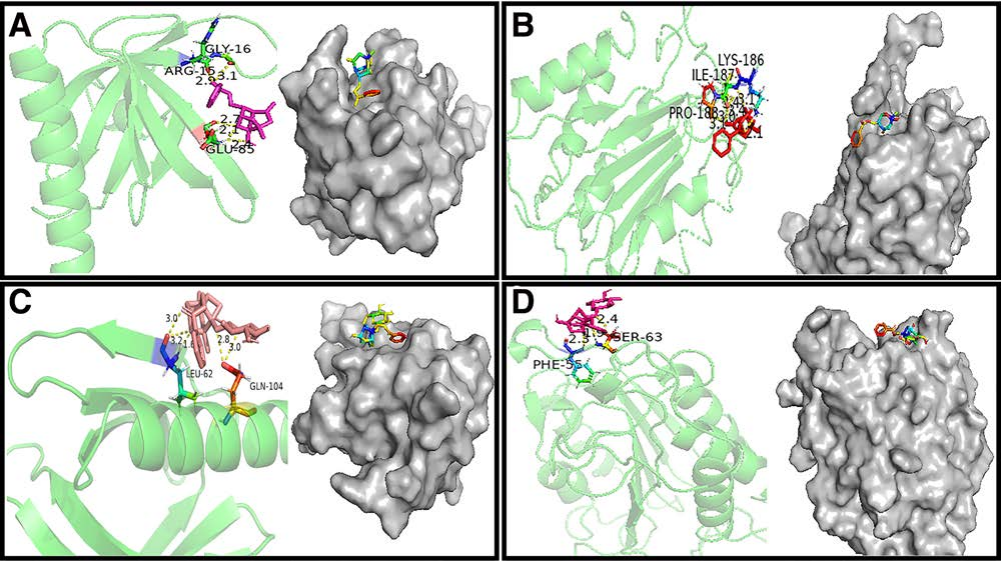
The hydrogen bond lengths, amino acid residues, and the active pockets between active components and proteins. (A) Paeoniflorgenone-AKT1. (B) Paeoniflorgenone-CASP3. (C) Paeoniflorin-AKT1. (D). Paeoniflorin-CASP3.

The 2 key components with a higher degree (paeoniflorgenone and paeoniflorin) successfully docked with the 2 core targets with a higher degree (AKT1 and CASP3), respectively. Negative binding energy was the premise for successful docking. In general, the lower the binding energy of ligand and receptor is, the more stable the binding is. The key components of RPA had a high affinity for the core targets (Table 8). The area where the components bind to the protein is called the active pocket. The molecular docking results and the active pockets were visualized using PyMoL software (Fig. 6).

**Table 8:** The genes involved in the top 10 Kyoto Encyclopedia of Genes and Genomes signaling pathways.

ID	Pathways	Gene names
hsa05417	Lipid and atherosclerosis	AKT1 CASP3 CASP7 MAPK14 CYP2C9 GSK3B HSPA8 HSP90AA1 PPARG MAPK1 MAPK8 MAPK10
hsa05215	Prostate cancer	AKT1 AR EGFR GSK3B GSTP1 HSP90AA1 IGF1R MAPK1
hsa01522	Endocrine resistance	AKT1 MAPK14 EGFR ESR1 IGF1R MAPK1 MAPK8 MAPK10
hsa04917	Prolactin signaling pathway	AKT1 MAPK14 ESR1 GSK3B MAPK1 MAPK8 MAPK10
hsa05145	Toxoplasmosis	AKT1 CASP3 MAPK14 HSPA8 PIK3CG MAPK1 MAPK8 MAPK10
hsa05210	Colorectal cancer	AKT1 CASP3 EGFR GSK3B MAPK1 MAPK8 MAPK10
hsa04657	IL-17 signaling pathway	CASP3 MAPK14 GSK3B HSP90AA1 MAPK1 MAPK8 MAPK10
hsa04914	Progesterone-mediated oocyte maturation	AKT1 MAPK14 HSP90AA1 IGF1R MAPK1 MAPK8 MAPK10
hsa04932	Nonalcoholic fatty liver disease	AKT1 CASP3 CASP7 MAPK14 GSK3B PPARG MAPK8 MAPK10
hsa05120	Epithelial cell signaling in Helicobacter pylori infection	CASP3 MAPK14 EGFR MAPK8 MAPK10 ADAM17

## 4. Discussion

The pathogenesis of RLS still remains unclear. It is often considered to be associated with dopaminergic dysfunction in the central nervous system, iron deficiency, peripheral nerves, vascular diseases, oxidative stress, and immunological abnormality.^[24–28,42–57]^ Weinstock et al^[28]^ studied many RLS-related diseases and found that 95% of the 38 diseases which were significantly correlated with RLS had changes in inflammation and immunity. Furthermore, they speculated that inflammation might cause iron deficiency and induce RLS. The pathogenesis of RLS tends to be complicated, making it difficult for single-target medications to obtain better therapeutic effects. The exploration of treatment with multi-targets and multi-pathways is the common goal of traditional Chinese and western medicine. TCM believes that the basic pathogenesis of RLS is based on the lack of nourishment of yin and blood in tendons. RPA can nourish yin and blood while further relieving the discomfort of the tendon. It is frequently used in the clinical treatment of RLS.

In this study, several key active components of RPA in treating RLS were screened, including paeoniflorin and paeoniflorgenone. TGP was involved in immune regulation, anti-inflammatory effect, brain protection, and nerve protection.^[18]^ Paeoniflorin has a wide range of anti-inflammatory and immunomodulatory effects.^[58]^ It could restore the downregulation of dopamine D2 receptor protein expression in the pituitary and hypothalamus induced by olanzapine^[59]^ and was a neuroprotective monoterpene glycoside with a good antidepressant effect. The mechanism was linked to upregulating the levels of monoaminergic neurotransmitters, inhibiting the hyperfunction of the hypothalamic-pituitary-adrenal axis, promoting neuroprotection and hippocampus neurogenesis, upregulating brain-derived neurotrophic factor level, inhibiting inflammatory reaction, and downregulating nitric oxide level.^[60]^ Paeoniflorgenone was a depolarization neuromuscular blocker similar to succinic choline, but it did not produce any contraction, while succinic choline did.^[61]^

In this study, we obtained several core targets that might be critical in the mechanism of RPA in the treatment of RLS, such as ALB, AKT1, CASP3, and others. Iron deficiency in the brain was associated with the pathophysiology of RLS.^[26,42,44–47,51,52]^ Serum ALB might interact with other serum factors (such as transferrin) to limit the supply of iron, thereby limiting the growth of invasive microorganisms.^[62]^ Changes in the dopaminergic system caused by iron deficiency might lead to RLS.^[53,54]^ The immune response to gastrointestinal bacteria or other antigens might cause RLS through direct immune attacks on the central or peripheral nervous system.^[28]^ AKT disorders could lead to neurological diseases.^[63]^ CASP3,^[64,65]^ EGFR,^[66]^ and AR^[67]^ were associated with the pathogenesis of nervous system diseases and could be involved in the regulation of the central nervous system.

The KEGG enrichment results showed that the possible pathways involved in the treatment process included lipid and atherosclerosis, endocrine resistance, prolactin signaling pathway, and IL-17 signaling pathway. Oxidative stress might participate in the pathogenesis of RLS.^[27,55–57]^ PPARG, one of the core targets, was confirmed to be involved in lipid metabolism and oxidative stress.^[68–70]^ IGF1R was also associated with oxidative stress.^[71]^ The pathogenesis of RLS was associated with the endocrine system.^[72]^ AR,^[73]^ ESR1,^[74]^ EGFR,^[75]^ and IGF1R^[76]^ were involved in endocrine regulation. The prolactin signaling pathway was closely related to dopamine function. IL-17 was an inflammatory cytokine mainly produced by CD4^+^ T cells that played an important role in the pathogenesis of immune disorders.^[77]^ HSP90AA1, MAPK1, MAPK8, and MAPK14 in the core targets were related to the IL-17 signaling pathway. HSP90AA1 was one of the proteins in the IL-17 signaling pathway.^[78]^ MAPK signaling pathway was downstream of the IL-17 signaling pathway. Therefore, RPA may be used to treat RLS via the aforementioned pathways.

## 5. Conclusion

RLS studies were mostly carried out in clinics. Few RLS studies involved animal or cell experiments, and the ideal animal model of RLS is still under exploration.^[79,80]^ Therefore, further experiments were not carried out in this study. Network pharmacology and molecular docking are rarely used to study RLS. However, based on network pharmacology and molecular docking, this study analyzed the possible mechanism of RPA in treating RLS with multiple components, multiple targets, and multiple pathways, which laid the foundation for future research, making this study innovative.

## Author contributions

**Conceptualization:** Jun Liu, Shengkai Mu, Suxian Liu, Tengteng Wang.

**Data curation:** Jun Liu, Shengkai Mu, Liansheng Hao, Fangfang Liu.

**Investigation:** Jun Liu, Shengkai Mu.

**Methodology:** Jun Liu, Shengkai Mu, Liansheng Hao, Fangfang Liu.

**Project administration:** Jun Liu.

**Resources:** Jun Liu, Shengkai Mu.

**Software:** Jun Liu, Shengkai Mu.

**Visualization:** Jun Liu, Shengkai Mu.

**Writing – original draft:** Jun Liu, Shengkai Mu.

**Writing – review & editing:** Suxian Liu, Tengteng Wang.
